# Cation, Anion and Ion-Pair Complexes with a G-3 Poly(ethylene imine) Dendrimer in Aqueous Solution

**DOI:** 10.3390/molecules22050816

**Published:** 2017-05-16

**Authors:** Matteo Savastano, Carla Bazzicalupi, Claudia Giorgi, Paola Gratteri, Antonio Bianchi

**Affiliations:** 1 Department of Chemistry “Ugo Schiff”, via della Lastruccia 3, 50019 Sesto Fiorentino, Italy; matteo.savastano@unifi.it (M.S.); carla.bazzicalupi@unifi.it (C.B.); Claudia.giorgi@unifi.it (C.G.); 2 NEUROFARBA Department, Pharmaceutical and Nutraceutical Section, and Laboratory of Molecular Modeling Cheminformatics & QSAR, University of Florence, Via Ugo Schiff 6, 50019 Sesto Fiorentino, Italy; paola.gratteri@unifi.it

**Keywords:** copper, zinc, dendrimers, poly(ethylene imine), polynuclear complexes, anion complexes, ion-pair complexes

## Abstract

The G-3 poly(ethylene imine) ligand L2 shows a multifaceted coordination ability, being able to bind metal cations, anions and ion-pairs. The equilibrium constants for the formation of metal (Cu^2+^, Zn^2+^), anion (SO_4_^2−^) and ion-pair (Cu^2+^/SO_4_^2−^) complexes were determined in 0.1 M Me_4_NCl aqueous solution at 298.1 ± 0.1 K by means of potentiometric titrations. Thanks to its dendrimeric nature, L2 can form highly nucleated metal complexes, such as Cu_5_L2^10+^ and Zn_4_L2^8+^, in successive and well-defined complexation steps. Protonated forms of L2 give rise to relatively weak anion complexes with SO_4_^2−^, but the addition of Cu^2+^ significantly enhances the binding ability of the ligand toward this anion below pH 9. In more alkaline solutions, an opposite trend is observed. The coordination properties of L2 are discussed with the support of modelling calculations. According to results, L2 is a promising molecule for the preparation of solid supported materials for the recovery of cations and anions from aqueous media and/or for applications in heterogeneous catalysis.

## 1. Introduction

**Figure 1:**
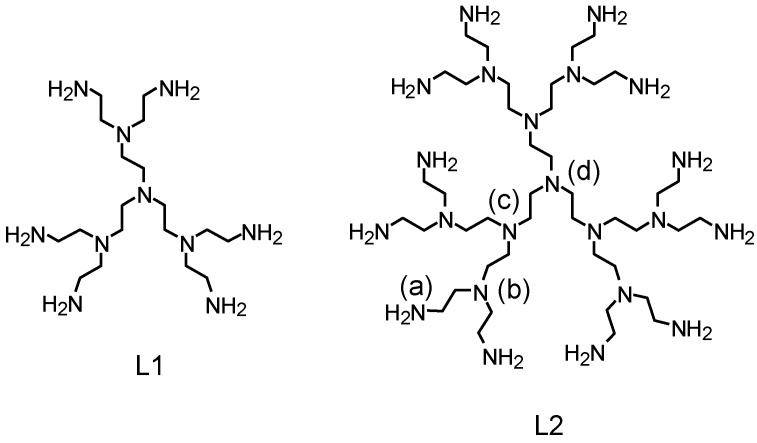
G-2 (**L1**) and G-3 (**L2**) poly(ethylene imine) dendrimers.

In recent papers, we showed that the G-2 poly(ethylene imine) dendrimer L1 (Figure 1) and its variously protonated forms are able to assemble stable cation, anion and ion-pair complexes in aqueous solution [1,2,3,4]. Such ability appears to be a propagation and an enhancement of the properties of the parent ligand tris(2-aminoethyl)amine (*tren*), which is historically known to bind metal complexes and, more recently, has also been accredited as a rather efficient anion receptor [5]. Indeed, regarding the coordination of metal ions, while *tren* forms stable mononuclear complexes, L1 can bind two metal ions such as Ni^2+^, Zn^2+^ and Cd^2+^ and up to three Cu^2+^ and Hg^2+^ ions [1,4]. L1 and its metal complexes are also able to bind inorganic anions [2] as well as the anionic forms of AMP, ADP and ATP nucleotides acting as catalysts that enhance significantly ATP dephosphorylation in aqueous solution [3]. Construction of a third generation of ethylamino branches around L1 gave rise to the G-3 poly(ethylene imine) dendrimer L2 (Figure 1) that is also able to bind anions, such as PO_4_^3−^, P_2_O_7_^4−^ and P_3_O_10_^5−^, and AMP, ADP and ATP nucleotides. In particular, L2 showed an unprecedented behaviour toward ATP, the dendrimer being able to enhance or inhibit dephosphorylation of the nucleotide depending on the solution pH [6]. 

Despite the discovery of such properties towards nucleotide and phosphate type anions, the ability of L2 to bind metal cations, inorganic anions and ion-pairs remains unexplored. Actually, L2 is a very interesting ligand for the formation of metal complexes, in particular of polynuclear assemblies, since it contains a large number (22) of nitrogen donor atoms in its molecular structure, and accordingly, it should be able to form complexes of greater nuclearity than the smaller homologous L1. It is worth of note that there is a great deal of interest toward polynuclear metal complexes, especially for their catalytic properties and/or for their use in the generation of metal nanoparticle-based catalysts [7,8,9,10].

This has aroused our interest in performing a detailed analysis of the complexation equilibria involving L2, metal cations and anions in aqueous solution. As previously noted [1], to study similar complexation systems is an arduous task, due to the many equilibria involving the stepwise coordination of several metal ions involving several protonation states for each complexation step. Attempts to perform the speciation of complex systems and determining the equilibrium constants for complexation equilibria with other poly(ethylene imine) dendrimers were made by considering the repeating triamine units of the dendrimer as identical independent ligand molecules, under the implicit assumption that these repeating units were uniformly distributed in solution, in contrast to their actual localization within the same dendrimer molecule [11]. These studies were performed under conditions approaching the ligand coordinative saturation, the metal-to-triamine unit ratios being closed to 1:1 and extending it, at most, up to 1:4.

Despite such an approximation, the results of these studies can be functional for the purposes for which they are developed, although they furnish an incomplete picture of the complexation systems. In particular, this approach leads to the identification of a limited number of complex species relative to the many that these dendrimers can form. For instance, dendrimers containing large numbers of amino groups are expected to bind metal ions even when they are extensively protonated. Some of the missing species could have interesting properties, like the ability of highly protonated Zn(II) complexes with L1 to promote the binding and the dephosphorylation of ATP [3].

In this paper, we report the results of a detailed analysis of the complexation systems formed by L2 with Zn^2+^ and Cu^2+^ that led to the identification of 35 and 42 complex species for Zn^2+^ and Cu^2+^, respectively, the ligand achieving the stepwise coordination of 4 Zn^2+^ or 5 Cu^2+^ ions. Once these complexation systems were clearly defined, we analysed the ability of L2 to interact with SO_4_^2−^ both in the absence and in the presence of Cu^2+^.

## 2. Results and Discussion

### 2.1. Formation of Metal Complexes

Speciation of L2/Cu^2+^ and L2/Zn^2+^ complex systems and determination of the relevant stability constants were performed by means of pH-metric (potentiometric) titrations (0.1 M Me_4_NCl, 298.1 ± 0.1 K) and analysis of the associated data by means of the computer program HYPERQUAD [12] which furnished the stability constants collected in Table 1 and Table 2 for Cu^2+^ and Zn^2+^, respectively. Distribution diagrams of the complexes formed are reported in Figures S1 and S2.

As shown by these tables, the G-3 dendrimer L2 is able to bind in successive steps from one to five Cu^2+^ cations and from one to four Zn^2+^ ions. According to the presence of many (22) nitrogen donor atoms in the ligand, all complexes but Cu_5_L2^10+^ are able to bind protons, and the number of protonated species they form decreases with increasing complex nuclearity. It was previously reported that protonation of the primary amine groups of L2 is associated with protonation constants log*K* ≥ 8.3 [6]. Considering this value as the limiting value for protonation of primary amine groups also in L2 complexes, we can deduce from the equilibrium data in Table 1 that, in CuL2^2+^, there are nine primary amine groups, out of 12, that are not involved in metal coordination. By similar reasoning, and taking into account the experimental errors on the determined equilibrium constants, the number of uncoordinated primary nitrogens can be reasonably estimated as six in Cu_2_L2^4+^, five in Cu_3_L2^6+^, three in Cu_4_L2^8+^, and none in Cu_5_L2^10+^. The equilibrium constants for the successive binding of the first and the second Cu^2+^ ions are very high (log*K* = 23.66 and 22.9, Table 1) and consistent with the stability of hexacoordinated Cu^2+^ complexes of polyamines [13,14]. Accordingly, the first two Cu^2+^ ions binding L2 should be coordinated by three primary and three secondary amine groups near the surface of the G-3 dendrimer. The third coordination stage causes a greater involvement of the inner dendrimer region, since only one primary amine group is involved in the equilibrium Cu_2_L2^4+^ + Cu^2+^ = Cu_3_L2^6+^. This appears to be a poorly favourable coordination step as shown by the surprisingly low value of the corresponding equilibrium constant (log*K* = 10.0, Table 1).

Such a drop of the metal ion binding constant is, most likely, determined by an important structural rearrangement that the very stable Cu_2_L2^4+^ complex must bear to accommodate the third Cu^2+^ ion. Conversely, Cu_3_L2^6+^ displays a greater binding ability toward Cu^2+^ than Cu_2_L2^4+^ (Cu_3_L^6+^ + Cu^2+^ = Cu_4_L^8+^, log*K* = 16.0, Table 1), that is, the coordination of the third Cu^2+^ ion is not very favourable but generates the structural conditions for a favourable continuation of the stepwise binding process. Two of the 5 free primary amine groups of Cu_3_L2^6+^ become coordinated in Cu_4_L2^8+^, while no primary amine group appears to be available for protonation in Cu_5_L2^1^^0^^+^. The formation of the latter from the tetranuclear complex is accompanied by a small equilibrium constant (Cu_4_L^8+^ + Cu^2+^ = Cu_5_L^1^^0^^+^, log*K* = 9.4, Table 1) in agreement with the high electrostatic repulsion exerting between the five metal ions and the reduced number of donor atoms remaining available for coordination in Cu_4_L2^8+^. As a matter of fact, the ligand is not able to fulfil the coordination sphere of all five metal ions in Cu_5_L2^1^^0^^+^ and facile dissociation of coordinated water molecules generates the hydroxo complex [Cu_5_L2(OH)_2_]^8+^.

In contrast to Cu^2+^, in the case of Zn^2+^ complexation, the equilibrium constants for the successive binding of metal ions to form ZnL2^2+^, Zn_2_L2^4+^, Zn_3_L2^6+^ and Zn_4_L2^8+^ (log*K* = 17.8, 13.2, 11.0, 10.7, Table 2) display a more regular trend. The loss of stability from the mono- to the binuclear complex (log*K* = 17.8, 13.2, Table 2) is greater than for the corresponding equilibria with Cu^2+^. Nevertheless, also the stability constants for the formation of ZnL2^2+^ and Zn_2_L2^4+^ are consistent with the stability of hexacoordinated Zn^2+^ complexes with polyamines [15,16]. According to the criterium based on complex protonation constants, the number of uncoordinated primary amine group should be 9 in ZnL2^2+^ and 6 in Zn_2_L2^4+^, in agreement with a coordination sphere constituted by three primary and three tertiary nitrogen atoms for both metal ions. Binding of the third Zn^2+^ ion takes place with further decrease of stability (Zn_2_L2^4+^ + Zn^2+^ = Zn_3_L2^6+^, log*K* = 11.0, Table 2). At this stage, another three primary amine groups become involved in metal binding, suggesting a similar hexacoordination for all three metal ions in Zn_3_L2^6+^. An insignificant decrease of binding constant is instead observed at the fourth coordination step (Zn_3_L2^6+^ + Zn^2+^ = Zn_4_L2^8+^, log*K* = 10.7, Table 2) even though an important reorganization of the trinuclear complex must occur to accommodate the fourth Zn^2+^ ion. According to protonation data in Table 2, two primary nitrogen atoms should remain uncoordinated in Zn_4_L2^8+^.

**Figure 2:**
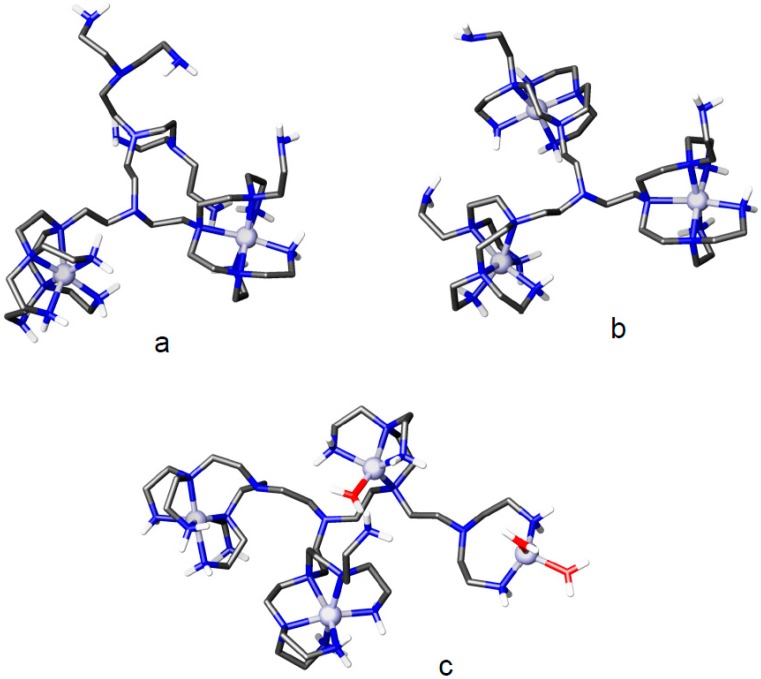
Minimum energy conformations calculated for (**a**) Zn_2_L2^4+^; (**b**) Zn_3_L2^6+^ and (**c**) Zn_4_L2^8+^.

To get insight into the structural properties of these Zn^2+^ polynuclear complexes, we performed molecular modelling calculations on Zn_2_L2^4+^, Zn_3_L2^6+^ and Zn_4_L2^8+^ in a simulated implicit water environment. The lower energy structures obtained for these complexes are shown in Figure 2. According to these structures, in Zn_2_L2^4+^ (Figure 2a) and Zn_3_L2^6+^ (Figure 2b) each metal ion is coordinated, in a distorted octahedral environment, to six nitrogen atoms pertaining to one arm of the ligand originating from the central tertiary amine group. In agreement with the deductions drawn above from the equilibrium constants, the number of primary nitrogen atoms remaining not coordinated is six in Zn_2_L2^4+^ (Figure 2a) and three in Zn_3_L2^6+^ (Figure 2b). Indeed, the addition of the fourth Zn^2+^ ion causes a major rearrangement of the trinuclear complex. The ligand displays a great ability to minimize the electrostatic repulsion between metal cations bringing them at long distance from each other (Figure 2c). Only one of the Zn^2+^ ions retains the octahedral coordination environment seen in the trinuclear complex, while the other three metal cations are: one pentacoordinated by ligand nitrogen atoms, one pentacoordinated by four ligand donors and a water molecule, one tetracoordinated by two ligand donors and two water molecules. The last coordination environment requires some cautionary considerations. In this complex unit, the ligand forms an 8-membered chelate ring including a not coordinated nitrogen atom. A similar arrangement is unlikely to occur in a real solution, since chelate rings of such size are poorly stable. In the simulated implicit water environment of our calculations, however, an overestimation of electrostatic repulsions could have forced the Zn^2+^ ion to stay as far as possible from the other three cations, instead of involving the third nitrogen atom in the formation of two stable 5-membered chelate rings, which is the situation that we expect to occur in water. Nevertheless, the calculated structure of Zn_4_L2^8+^ (Figure 2c) seems very representative of the overall organization of this complex, as shown by the fact that it implicates the presence of two not coordinated primary nitrogen atoms in agreement with the results deduced above from equilibrium data.

### 2.2. Formation of Anion and Ion-Pair Complexes

The detailed analysis of metal complexation equilibria with L2 makes it possible to further investigate such equilibrium systems. For instance, it is possible to analyse the ability of L2 complexes to interact with other species in the environment. We have already seen that protonated forms of L2 can bind PO_4_^3−^, P_2_O_7_^4−^, P_3_O_10_^5−^, and nucleotides (AMP, ADP, ATP) anions in solution [6], and we have already seen that the G-2 dendrimer L1 is able to form both anion and ion-pair complexes [2,3]. We have now studied the equilibria involving L2 and SO_4_^2−^ both in the absence and in the presence of Cu^2+^ ions by means of pH-metric (potentiometric) titrations (0.1 M Me_4_NCl, 298.1 ± 0.1 K). Indeed, the potentiometric data, treated with the computer program HYPERQUAD [12], revealed that many protonated forms of L2 are able to bind both the SO_4_^2−^ anion alone and the Cu^2+^/SO_4_^2−^ ion-pair. The equilibrium constants for the formation of SO_4_^2−^ complexes are reported in Table 3 (see Figure S3 for a distribution diagram). This table includes the overall constants (*β* values) for the binding of SO_4_^2−^ along with the constants for the equilibria of anion binding by protonated ligand species (H*_n_*L2*^n^*^+^ + SO_4_^2−^ = [H*_n_*L2(SO_4_)]^(*n*−2)+^) that could be calculated from the former by using the ligand protonation constants [6]. It is to be noted that the constants for the latter equilibria could not be calculated for complexes [H*_n_*L2(SO_4_)]^(*n*−2)+^ with *n* < 11 (Table 3), since it was not possible to resolve as single proton binding processes the protonation equilibria involving H*_n_*L2*^n^*^+^ species with *n* < 11 [6]. Nonetheless, the stability constants that are available for the binding of SO_4_^2−^ to the protonated ligand forms show some peculiarities of this ligand. The stability of anion complexes of polyammonium ligands is generally determined by electrostatic attraction and hydrogen bonding [17,18,19,20,21]. Conversely, the ability of L2 to bind SO_4_^2−^ appears to be unaffected by its positive charge, that is, by its protonation state. Actually, the equilibrium constants for the anion binding vary in a very reduced range and their values are very small, on consideration of the high positive ligand charge and in comparison with SO_4_^2−^ complexes of other polyammonium ligands [22]. A similar behaviour was also observed for phosphate and phosphate-like anion complexes with L2, although, in several cases, the stability of these complexes was significantly higher, probably due to the greater hydrogen bond ability of phosphate-like anions [6]. Also the trend of stability is particular: the stability constants decrease from H_11_L2^11+^ (log*K* = 3.10) to H_13_L2^13+^ (log*K* = 2.46), then steadily increase up to the formation of the complex with H_18_L2^18+^ (log*K* = 3.32).

**Figure 3:**
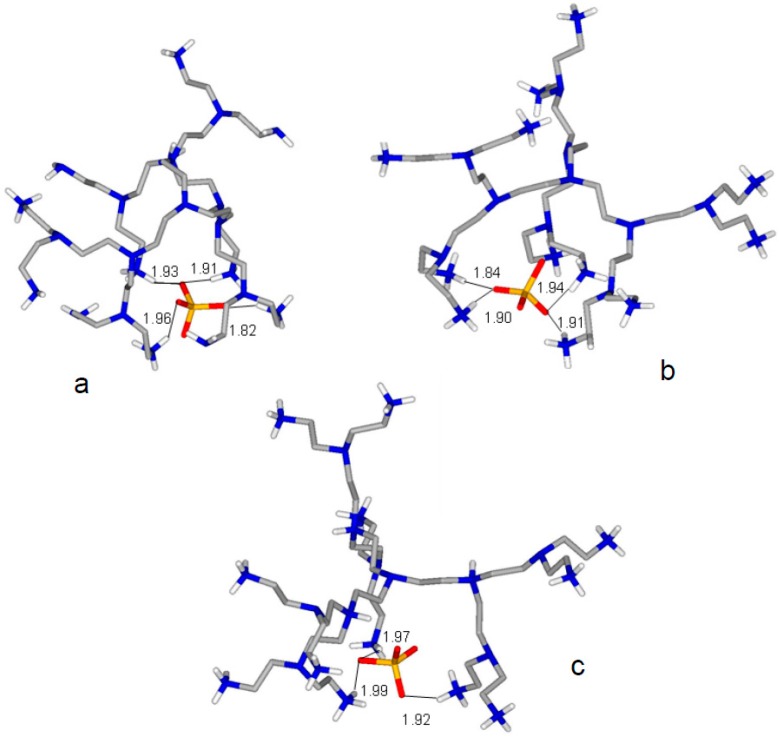
Minimum energy conformations calculated for (**a**) [H_6_L2(SO_4_)]^4+^; (**b**) [H_12_L2(SO_4_)]^10+^ and (**c**) [H_15_L2(SO_4_)]^13+^. Distances are in Å.

To get information about the possibility that such behaviour originates form the structural characteristics of the anion complexes, we performed a molecular modelling calculation on the [H_6_L2(SO_4_)]^4+^, [H_12_L2(SO_4_)]^1^^0^^+^ and [H_15_L2(SO_4_)]^13+^ species, assuming that the localization of H^+^ ions in the protonated ligand forms is as previously established by ^1^H-NMR spectroscopy [6], that is, the first 12 H^+^ ions bind the 12 primary N(a) atoms (Figure 1), while in H_15_L2^15+^ the three additional protons involve the three tertiary N(c) nitrogen atoms. In [H_6_L2(SO_4_)]^4+^, protonation was assumed to occur on primary amine groups located as far apart as possible from each other. The minimum energy structures calculated for these complexes, reported in Figure 3, show that the ligand molecule becomes increasingly expanded while becoming increasingly protonated, as a consequence of the increasing electrostatic repulsion exerting between the ammonium groups. In the minimum energy structures of [H_6_L2(SO_4_)]^4+^ (Figure 3a) and [H_12_L2(SO_4_)]^1^^0^^+^ (Figure 3b), the SO_4_^2−^ anion forms four salt-bridges (charge reinforced hydrogen bonds) with four ammonium groups of the ligand, while in [H_15_L2(SO_4_)]^13+^ (Figure 3c) such interactions drop to three and become longer. Most likely, the two opposite trends developing with increasing ligand protonation, namely (i) the favourable contribution due to the increasing ligand charge; (ii) the unfavourable contribution determined by ligand expansion, are responsible for the particular trend of complex stability showing a minimum for SO_4_^2−^ binding by H_13_L2^13+^ (log*K* = 2.46, Table 3).

As anticipated above, L2 can bind SO_4_^2−^ and Cu^2+^, simultaneously, forming ion-pair complexes. The equilibrium constants determined for such complexes are presented in Table 4 in the form of equilibrium constants for SO_4_^2−^ binding the by Cu^2+^ complexes of L2 (see Figure S4 for a distribution diagram). The analysis of the L2/Cu^2+^/SO_4_^2−^ system was limited to the formation of ion-pair complexes containing a single metal ion (see the experimental section). Nevertheless, even under the appropriate conditions, the [Cu_2_L2(SO_4_)]^2+^ complex was also found (Table 4), evidencing that more complex ion-pair species including more than one Cu^2+^ ion can be formed in solution. However, the analysis of such systems, requiring consideration of more than 92 equilibria, did not produced univocal results. This is the reason why we limited our study to ion-pair complexes with a single metal ion.

**Figure 4:**
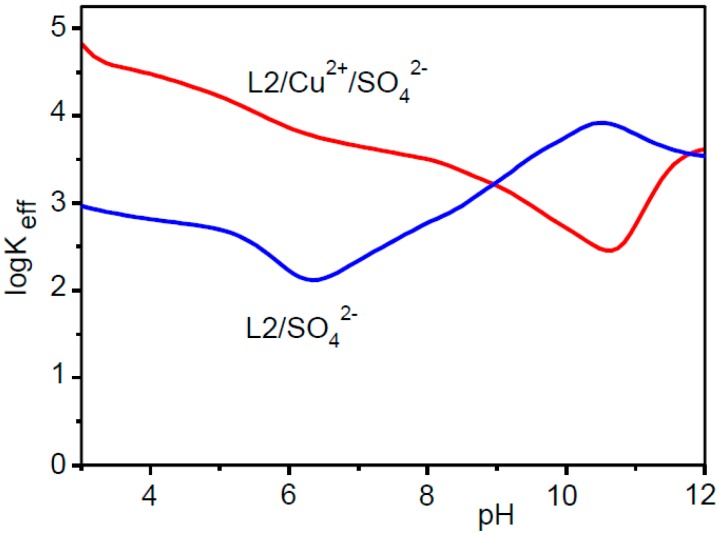
Logarithms of the conditional stability constants of anion (SO_4_^2−^) and ion-pair (Cu^2+^/SO_4_^2−^) complexes with L2.

As can be seen from Table 4, the ability of the protonated Cu^2+^ complexes to bind SO_4_^2−^ increases almost steadily with the positive charge of the metal complex, that is with its protonation state, the unique exception being represented by [CuH_10_L2(SO_4_)]^1^^0^^+^, whose formation constant appears to be a little bit smaller than that of [CuH_9_L2(SO_4_)]^9+^. An assessment of the ability of the ligand to bind the anion in the absence or in the presence of Cu^2+^ ions can be performed by direct comparison of the equilibrium constants in Table 3 and Table 4, limited to species with H_11_L2^11+^ to H_18_L2^18+^ ligand forms. Such comparison shows that the presence of Cu^2+^ enhances the ability of these ligand species to bind SO_4_^2−^, the increment growing with increasing ligand protonation. For instance, if we consider SO_4_^2−^ binding by species with equal positive charge, such as H_18_L2^18+^ and CuH_16_L2^18+^, we observed an increase in stability from log*K* = 3.32 (H_18_L2^18+^ + SO_4_^2−^ = [H_18_L2(SO_4_)]^16+^) to log*K* = 5.20 (CuH_16_L2^18+^ + SO_4_^2−^ = [CuH_16_L2(SO_4_)]^16+^), corresponding to a free energy increment of 11 kJ/mol. For ligand species in lower protonation state than H_11_L2^11+^, a similar comparison cannot be performed due to the already mentioned impossibility of expressing in the form H_n_L2*^n^*^+^ + SO_4_^2−^ = [H*_n_*L2(SO_4_)]^(*n*−2)+^ the formation constants of [H*_n_*L2(SO_4_)]^(*n*−2)+^ complexes with *n* < 11. To overcome this problem, we can make use of the so called conditional (effective) stability constants that can be calculated for each system, as a function of pH, in the form *K*_eff_ = Σ[AH*_i_*L]/(Σ[H*_j_*L] × [A]), for anion complexes (A = SO_4_^2−^), and *K*_eff_ = Σ[CuAH*_k_*L]/(Σ[CuH*_l_*L] × [A]), for ion-pair complexes, where *i*, *j*, *k* and *l* are the number of acidic protons on the ligand in the different species [23]. As can be seen from Figure 4, which shows the variation with pH of the effective stability constants calculated for SO_4_^2−^ and ion-pair complexes, the presence of Cu^2+^ promotes the binding of SO_4_^2−^ below pH 9, while in the range 9 < pH < 10.5 there is a preference for the metal-free ligand. This behaviour suggests the involvement of the metal ion in the binding of SO_4_^2−^ in the ion-pair complexes of higher protonation state. At high pH values, the ligand is poorly protonated and thus it is able to fulfil the coordination sphere of Cu^2+^, preventing metal coordination to SO_4_^2−^. The ligand wraps around the metal ion leaving less space for SO_4_^2−^. Upon protonation of the Cu^2+^ complex, the ligand becomes less involved in the coordination to the metal and the increasing positive charge of the complex expands its structure, thus making space for the anion to get in contact with Cu^2+^ and form an increasing number of salt-bridges with ligand ammonium groups. At the break point of these trends (pH 9), the main ion-pair species in solution is [CuH_7_L2(SO_4_)]^7+^ (Figure S4). Below pH 6.5, the separation between the two curves in Figure 4, becomes about 2 logarithm units, which corresponds to the 11 kJ/mol free energy increment observed above for the binding of SO_4_^2−^ to CuH_16_L2^18+^ relative to H_18_L2^18+^. The formation of contact ion-pair complexes was previously reported for the G-2 dendrimer L1, and is corroborated for L2 by the fact that the binuclear Cu_2_L2^4+^ complex binds SO_4_^2−^ (Table 4) in the absence of ligand ammonium groups (ligand protonation).

## 3. Materials and Methods

### 3.1. General Information

All starting materials were high purity compounds purchased from commercial sources and used as supplied. Ligand L2 was synthetized according to a previously described procedure [24].

### 3.2. Potentiometric Measurements

Potentiometric (pH-metric) titrations, employed to determine equilibrium constants, were performed in 0.1 M Me_4_NCl aqueous solution at 298.1 ± 0.1 K by using an automated system and a procedure already described [25]. The combined Metrohm 6.0262.100 electrode (Metrohm AG, Herisau, Switzerland) was calibrated as a hydrogen-ion concentration probe by titration of previously standardized amounts of HCl with CO_2_-free NMe_4_OH solutions and determining the equivalent point by Gran’s method [26], which gives the standard potential, *E*°, and the ionic product of water (p*K*_w_ = 13.83 (1) in 0.1 M Me_4_NCl at 298.1 ± 0.1 K). The computer program HYPERQUAD [12] was used to calculate complex stability constants. All experiments were performed in the pH range 2.5–11.0 with 1 × 10^−3^ M ligand concentration. Six titrations in the case of Cu^2+^ complexation, and five in the case of Zn^2+^, were performed with metal concentration varying in the ranges 0.5[L] ≤ [Cu^2+^] ≤ 4.5[L] and 0.5[L] ≤ [Zn^2+^] ≤ 3.5[L]. Metal to ligand molar ratios greater than 5 for Cu^2+^ and 4 for Zn^2+^ were also tested: precipitation of metal hydroxide was observed in alkaline solution, while the analysis of the acidic branches of the titrations confirmed the maximum nuclearity of 5 for Cu^2+^ and 4 for Zn^2+^. Three titrations were performed for anion binding with SO_4_^2−^ concentration in the range 2[L] ≤ [SO_4_^2−^] ≤ 5[L]. Three titrations were performed for ion-pair binding with [Cu^2+^] = 0.8[L] and SO_4_^2−^ concentration 2[L] ≤ [SO_4_^2−^] ≤ 5[L]. The different titration curves, obtained for metal, anion and ion-pair complexation experiments, respectively, were treated as separated curves without significant variations in the values of the common stability constants. Finally, the sets of data were merged together and treated simultaneously to give the final stability constants. Different equilibrium models for the complex systems were generated by eliminating and introducing different species. Only those models for which the HYPERQUAD program furnished a variance of residuals σ^2^ ≤ 9 were accepted. This condition was unambiguously met by a single model. Ligand protonation constants were taken from the literature [6].

### 3.3. Molecular Modelling

Molecular modelling investigations on [H_6_L2(SO_4_)]^4+^, [H_12_L2(SO_4_)]^1^^0^^+^ and [H_15_L2(SO_4_)]^13+^ complexes were performed by means of the empirical force field method AMBER3 as implemented in the Hyperchem 7.51 package [27], using an implicit simulation of aqueous environment (ε = 4 r) and atomic charged evaluated at the semiempirical level of theory (PM3) [28,29]. Potential energy surface of all the systems were explored by means of simulated annealing (T = 600 K, equilibration time = 10 ps, run time = 10 ps and cooling time = 10 ps, time step = 1.0 fs). For each studied system, 80 conformations were sampled.

As for the Zn(II) complexes, the trinuclear Zn_3_L2^6+^ species was firstly analysed. Starting coordinates were built from the crystal structure of the Ni^2+^ complex of L1 [1], containing Ni^2+^ ions hexacoordinated in distorted octahedral environments to six out of the seven nitrogen atoms constituting a portion of L1 that is identical to the three branches of L2 growing from the central N(c) atom (Figure 1). The nitrogen atom remaining uncoordinated is a primary one. This structural motif was chosen taking into account that, according to the equilibrium data discussed before, all three metal ions of Zn_3_L2^6+^ should be hexacoordinated and three primary amine groups of the complex should not be involved in metal coordination. This crystallographic structural unit was firstly modified by replacing Ni^2+^ with Zn^2+^ and completing each coordination environment with water molecules.

The starting coordinates for the binuclear Zn_2_L2^4+^ complex were obtained by deleting one zinc ion in the QM minimized structure of Zn_3_L2^6+^. The tetranuclear Zn_4_L2^8+^ complex was instead obtained from the QM minimized binuclear complex by adding to its metal-free branch two QM minimized pentacoordinated Zn^2+^ complexes, one in square pyramidal and one in bipyramidal geometry, taken from the crystallographic structure of the trinuclear Cu^2+^ complex of L1 [1] and successively modified by replacing Cu^2+^ with Zn^2+^.

The starting coordinates for each polynuclear complexes were firstly optimized by using the OPLS2005 forcefield implemented in the Impact software [30], with completely frozen metals and coordination environments. Then, each MM minimized structure was fully optimized at the DFT/M06 level of theory [31,32] by using the 6–31 g(tm) basis set [33,34,35,36,37] and the implicit simulation for the aqueous environment [38]. The nature of stationary points as true minima was checked by frequency calculations.

## 4. Conclusions

The ability of L2 to form stable highly nucleated complexes over a large pH range, as a consequence of its dendrimeric nature and of the many amine groups in its structure, make this compound a promising candidate for the preparation of solid supported materials to be used in the recovery of metal ions from aqueous media. This could find applications in both decontamination of waste waters and in the extraction of precious metals. Indeed, it was recently reported that activated carbon functionalized with randomly structured poly(ethylene imine) dendrimers are efficient scavenger of Pd^2+^ cations [39]. Moreover, L2 is also a promising candidate for catalytic purposes. The use of molecules with well-defined molecular structures, such as L2, has the advantage that with such molecules it is possible to perform a confident speciation of the complexes they form in solution, thus getting a fundamental instrument for the tailoring of appropriate receptors for substrates binding and activation. This is of special interest when the supported complex is used for catalytic purposes. Considering the ability of L2 to form complexes with many metal centres that may promote the binding of further species from the medium, we are particularly interested in developing carbon materials (activated carbons, carbon nanotubes, graphene) functionalized with L2 and testing them for catalytic applications in reaction for the formation of carbon-carbon bonds, such as the Sonogashira cross coupling.

## Figures and Tables

**Table 1:** Stability constants of Cu^2+^ complexes with L2. 0.1 M Me_4_NCl, 298.1 ± 0.1 K. Values in parentheses are standard deviation on the last significant figure.

Equilibria	log*K*	Equilibria	log*K*
Cu^2+^ + L2 = CuL2^2+^	23.66 (5)	Cu_2_H_7_L2^11+^ + H^+^ = Cu_2_H_8_L2^12+^	7.46 (8)
CuL2^2+^ + 2H^+^ = CuH_2_L2^4+^	22.88 (7)	Cu_2_H_8_L2^12+^ + H^+^ = Cu_2_H_9_L2^13+^	6.25 (8)
CuH_2_L2^4+^ + H^+^ = CuH_3_L2^5+^	9.93 (5)	Cu_2_H_9_L2^13+^ + H^+^ = Cu_2_H_10_L2^14+^	4.98 (7)
CuH_3_L2^5+^ + H^+^ = CuH_4_L2^6+^	10.07 (5)	Cu_2_H_10_L2^14+^ + H^+^ = Cu_2_H_11_L2^15+^	4.07 (7)
CuH_4_L2^6+^ + H^+^ = CuH_5_L2^7+^	9.42 (3)		
CuH_5_L2^7+^ + H^+^ = CuH_6_L2^8+^	9.21 (7)	3Cu^2+^ + L2 = Cu_3_L2^6+^	56.55 (7)
CuH_6_L2^8+^ + H^+^ = CuH_7_L2^9+^	9.09 (7)	Cu_2_L2^4+^ + Cu^2+^ = Cu_3_L2^6+^	10.0 (1)
CuH_7_L2^9+^ + H^+^ = CuH_8_L2^10+^	8.63 (5)	Cu_3_L2^6+^ + 2H^+^ = Cu_3_H_2_L2^8+^	22.72 (6)
CuH_8_L2^10+^ + H^+^ = CuH_9_L2^11+^	8.50 (4)	Cu_3_H_2_L2^8+^ + H^+^ = Cu_3_H_3_L2^9+^	10.31 (7)
CuH_9_L2^11+^ + H^+^ = CuH_10_L2^12+^	8.13 (4)	Cu_3_H_3_L2^9+^ + H^+^ = Cu_3_H_4_L2^10+^	8.87 (8)
CuH_10_L2^12+^ + H^+^ = CuH_11_L2^13+^	7.49 (4)	Cu_3_H_4_L2^10+^ + H^+^ = Cu_3_H_5_L2^11+^	8.50 (8)
CuH_11_L2^13+^ + H^+^ = CuH_12_L2^14+^	5.87 (4)	Cu_3_H_5_L2^11+^ + H^+^ = Cu_3_H_6_L2^12+^	7.39 (7)
CuH_12_L2^14+^ + H^+^ = CuH_13_L2^15+^	5.18 (4)	Cu_3_H_6_L2^12+^ + H^+^ = Cu_3_H_7_L2^13+^	6.82 (8)
CuH_13_L2^15+^ + H^+^ = CuH_14_L2^16+^	3.94 (5)	Cu_3_H_7_L2^13+^ + H^+^ = Cu_3_H_8_L2^14+^	5.81 (8)
CuH_14_L2^16+^ + H^+^ = CuH_15_L2^17+^	2.59 (5)		
CuH_15_L2^17+^ + H^+^ = CuH_16_L2^18+^	2.89 (6)	4Cu^2+^ + L2 = Cu_4_L2^8+^	72.6 (1)
		Cu_3_L2^6+^ + Cu^2+^ = Cu_4_L2^8+^	16.0 (1)
2Cu^2+^ + L2 = Cu_2_L2^4+^	46.53 (7)	Cu_4_L2^8+^ + 2H^+^ = Cu_4_H_2_L2^8+^	22.5 (1)
CuL2^2+^ + Cu^2+^ = Cu_2_L2^4+^	22.9 (1)	Cu_4_H_2_L2^10+^ + H^+^ = Cu_4_H_3_L2^11+^	8.54 (1)
Cu_2_L2^4+^ + H^+^ = Cu_2_HL2^5+^	11.51 (6)	Cu_4_H_3_L2^11+^ + H^+^ = Cu_4_H_4_L2^12+^	7.3 (1)
Cu_2_HL2^5+^ + H^+^ = Cu_2_H_2_L2^6+^	10.20 (7)	Cu_4_H_4_L2^12+^ + H^+^ = Cu_4_H_5_L2^13+^	6.9 (1)
Cu_2_H_2_L2^6+^ + H^+^ = Cu_2_H_3_L2^7+^	9.24 (7)	Cu_4_H_5_L2^13+^ + H^+^ = Cu_4_H_6_L2^14+^	3.9 (1)
Cu_2_H_3_L2^7+^ + H^+^ = Cu_2_H_4_L2^8+^	9.61 (6)		
Cu_2_H_4_L2^8+^ + H^+^ = Cu_2_H_5_L2^9+^	8.31 (7)	5Cu^2+^ + L2 = Cu_5_L2^10+^	82.0 (2)
Cu_2_H_5_L2^9+^ + H^+^ = Cu_2_H_6_L2^10+^	8.22 (7)	Cu_4_L2^8+^ + Cu^2+^ = Cu_5_L2^10+^	9.4 (3)
Cu_2_H_6_L2^10+^ + H^+^ = Cu_2_H_7_L2^11+^	8.18 (7)	Cu_5_L2^10+^ + 2OH^−^ =[Cu_5_L2(OH)_2_]^8+^	8.5 (2)

**Table 2:** Stability constants of Zn^2+^ complexes with L2. 0.10 M Me_4_NCl, 298.1 ± 0.1 K. Values in parentheses are standard deviation on the last significant figure.

Equilibria	log*K*	Equilibria	log*K*
Zn^2+^ + L2 = ZnL2^2+^	17.18 (5)	Zn_2_H_6_L2^10+^ + H^+^ = Zn_2_H_7_L2^11+^	8.13 (8)
ZnL2^2+^ + 2H^+^ = ZnH_2_L2^4+^	22.50 (8)	Zn_2_H_7_L2^11+^ + H^+^ = Zn_2_H_8_L2^12+^	7.36 (7)
ZnH_2_L2^4+^ + H^+^ = ZnH_3_L2^5+^	10.04 (5)	Zn_2_H_8_L2^12+^ + H^+^ = Zn_2_H_9_L2^13+^	6.47 (5)
ZnH_3_L2^5+^ + H^+^ = ZnH_4_L2^6+^	9.59 (6)		
ZnH_4_L2^6+^ + H^+^ = ZnH_5_L2^7+^	10.01 (7)	3Zn^2+^ + L2 = Zn_3_L2^6+^	41.36 (5)
ZnH_5_L2^7+^ + 2H^+^ = ZnH_7_L2^9+^	18.14 (7)	Zn_2_L2^4+^ + Zn^2+^ = Zn_3_L2^6+^	11.0 (1)
ZnH_7_L2^9+^ + H^+^ = ZnH_8_L2^10+^	8.25 (6)	Zn_3_L2^6+^ + 2H^+^ = Zn_3_H_2_L2^8+^	22.52 (6)
ZnH_8_L2^10+^ + H^+^ = ZnH_9_L2^11+^	8.64 (7)	Zn_3_H_2_L2^8+^ + H^+^ = Zn_3_H_3_L2^9+^	9.34 (8)
ZnH_9_L2^11+^ + H^+^ = ZnH_10_L2^12+^	7.97 (6)	Zn_3_H_3_L2^9+^ + H^+^ = Zn_3_H_4_L2^10+^	8.12 (8)
ZnH_10_L2^12+^ + H^+^ = ZnH_11_L2^13+^	6.92 (5)	Zn_3_H_4_L2^10+^ + H^+^ = Zn_3_H_5_L2^11+^	8.00 (8)
ZnH_11_L2^13+^ + H^+^ = ZnH_12_L2^14+^	5.75 (4)	Zn_3_H_5_L2^11+^ + H^+^ = Zn_3_H_6_L2^12+^	6.94 (6)
ZnH_12_L2^14+^ + H^+^ = ZnH_13_L2^15+^	5.38 (5)	Zn_3_H_6_L2^12+^ + H^+^ = Zn_3_H_7_L2^13+^	6.26 (6)
2Zn^2+^ + L2 = Zn_2_L2^4+^	30.35 (7)	4Zn^2+^ + L2 = Zn_4_L2^8+^	52.08 (8)
ZnL2^2+^ + Zn^2+^ = Zn_2_L2^4+^	13.2 (1)	Zn_3_L2^6+^ + Zn^2+^ = Zn_4_L2^8+^	10.7 (1)
Zn_2_L2^4+^ + H^+^ = Zn_2_HL2^5+^	11.27 (8)	Zn_4_L2^8+^ + H^+^ = Zn_4_HL2^9+^	9.48 (8)
Zn_2_HL2^5+^ + H^+^ = Zn_2_H_2_L2^6+^	11.44 (8)	Zn_4_HL2^9+^ + H^+^ = Zn_4_H_2_L2^10+^	8.90 (8)
Zn_2_H_2_L2^6+^ + H^+^ = Zn_2_H_3_L2^7+^	9.53 (8)	Zn_4_H_2_L2^10+^ + H^+^ = Zn_4_H_3_L2^11+^	8.24 (8)
Zn_2_H_3_L2^7+^ + H^+^ = Zn_2_H_4_L2^8+^	9.54 (8)	Zn_4_H_3_L2^11+^ + H^+^ = Zn_4_H_4_L2^12+^	7.35 (9)
Zn_2_H_4_L2^8+^ + H^+^ = Zn_2_H_5_L2^9+^	8.80 (9)	Zn_4_L2^8+^ + OH^−^ = [Zn_4_L2(OH)]^7+^	2.2 (1)
Zn_2_H_5_L2^9+^ + H^+^ = Zn_2_H_6_L2^10+^	8.60 (8)		

**Table 3:** Stability constants of the anion complexes formed by L2 with SO_4_^2−^. 0.1 M Me_4_NCl, 298.1 ± 0.1 K. Values in parentheses are standard deviation on the last significant figure.

Equilibria	log*K*	Equilibria	log*K*
L2 + 3H^+^ + SO_4_^2−^ = [H_3_L2(SO_4_)]^+^	38.09 (5)	H_11_L2^11+^ + SO_4_^2−^ = [H_11_L2(SO_4_)]^9+^	3.10 (7)
L2 + 5H^+^ + SO_4_^2−^ = [H_5_L2(SO_4_)]^3+^	57.88 (5)	H_12_L2^12+^ + SO_4_^2−^ = [H_12_L2(SO_4_)]^10+^	2.81 (7)
L2 + 7H^+^ + SO_4_^2−^ = [H_7_L2(SO_4_)]^5+^	76.63 (5)	H_13_L2^13+^ + SO_4_^2−^ = [H_13_L2(SO_4_)]^11+^	2.46 (7)
L2 + 9H^+^ + SO_4_^2−^ = [H_9_L2(SO_4_)]^7+^	94.62 (5)	H_15_L2^15+^ + SO_4_^2−^ = [H_15_L2(SO_4_)]^13+^	2.59 (7)
L2 + 11H^+^ + SO_4_^2−^ = [H_11_L2(SO_4_)]^9+^	111.54 (5)	H_16_L2^16+^ + SO_4_^2−^ = [H_16_L2(SO_4_)]^14+^	2.76 (7)
L2 + 12H^+^ + SO_4_^2−^ = [H_12_L2(SO_4_)]^10+^	119.58 (5)	H_17_L2^17+^ + SO_4_^2−^ = [H_17_L2(SO_4_)]^15+^	2.91 (7)
L2 + 13H^+^ + SO_4_^2−^ = [H_13_L2(SO_4_)]^11+^	127.24 (5)	H_18_L2^18+^ + SO_4_^2−^ = [H_18_L2(SO_4_)]^16+^	3.32 (7)
L2 + 15H^+^ + SO_4_^2−^ = [H_15_L2(SO_4_)]^13+^	139.90 (5)		
L2 + 16H^+^ + SO_4_^2−^ = [H_16_L2(SO_4_)]^14+^	145.53 (5)		
L2 + 17H^+^ + SO_4_^2−^ = [H_17_L2(SO_4_)]^15+^	149.44 (5)		
L2 + 18H^+^ + SO_4_^2−^ = [H_18_L2(SO_4_)]^16+^	152.12 (5)		

**Table 4:** Stability constants of the ion-pair complexes formed by L2 with Cu^2+^ and SO_4_^2−^. 0.1 M Me_4_NCl, 298.1 ± 0.1 K. Values in parentheses are standard deviation on the last significant figure.

Equilibria	log*K*
CuH_3_L2^5+^ + SO_4_^2−^ = [CuH_3_L2(SO_4_)]^3+^	3.10 (8)
CuH_5_L2^7+^ + SO_4_^2−^ = [CuH_5_L2(SO_4_)]^5+^	3.33 (5)
CuH_7_L2^9+^ + SO_4_^2−^ = [CuH_7_L2(SO_4_)]^7+^	3.51 (5)
CuH_9_L2^11+^ + SO_4_^2−^ = [CuH_9_L2(SO_4_)]^9+^	3.62 (5)
CuH_10_L2^12+^ + SO_4_^2−^ = [CuH_10_L2(SO_4_)]^10+^	3.44 (5)
CuH_11_L2^13+^ + SO_4_^2−^ = [CuH_11_L2(SO_4_)]^11+^	3.69 (5)
CuH_12_L2^14+^ + SO_4_^2−^ = [CuH_12_L2(SO_4_)]^12+^	3.96 (5)
CuH_13_L2^15+^ + SO_4_^2−^ = [CuH_13_L2(SO_4_)]^13+^	4.31 (5)
CuH_14_L2^16+^ + SO_4_^2−^ = [CuH_14_L2(SO_4_)]^14+^	4.64 (5)
CuH_16_L2^18+^ + SO_4_^2−^ = [CuH_16_L2(SO_4_)]^16+^	5.20 (5)
[CuH_16_L2(SO_4_)]^16+^ + H+ = [CuH_17_L2(SO_4_)]^17+^	2.78 (5)
Cu_2_L2^4+^ + SO_4_^2−^ = [Cu_2_L2(SO_4_)]^2+^	4.01 (5)

## Supplementary Materials

The following are available online: Figure S1: Distribution diagrams of the Cu^2+^ complexes of L2, Figure S2: Distribution diagrams of the Zn^2+^ complexes of L2, Figure S3: Distribution diagrams of the anion complexes formed by L2 with SO_4_^2−^, Figure S4: Distribution diagrams of the ion-pair complexes formed by L2 with Cu^2+^ and SO_4_^2−^ complexes of L2.
