# Supplementary material for: Cation, Anion and Ion-Pair Complexes with a G-3 Poly(ethylene imine) Dendrimer in Aqueous Solution
Source: Molecules. 2017 May 16;22(5):816. doi: 10.3390/molecules22050816 (PMC6154109; doi:10.3390/molecules22050816)
Supplement: Supplementary file 1 [file molecules-22-00816-s001.pdf]

Supplementary material

for

Cation, Anion and Ion-Pair Complexes with a G-3 Poly(ethylene imine) Dendrimer in Aqueous Solution.

By Matteo Savastano, Carla Bazzicalupi, Claudia Giorgi, Paola Gratteri and Antonio Bianchi

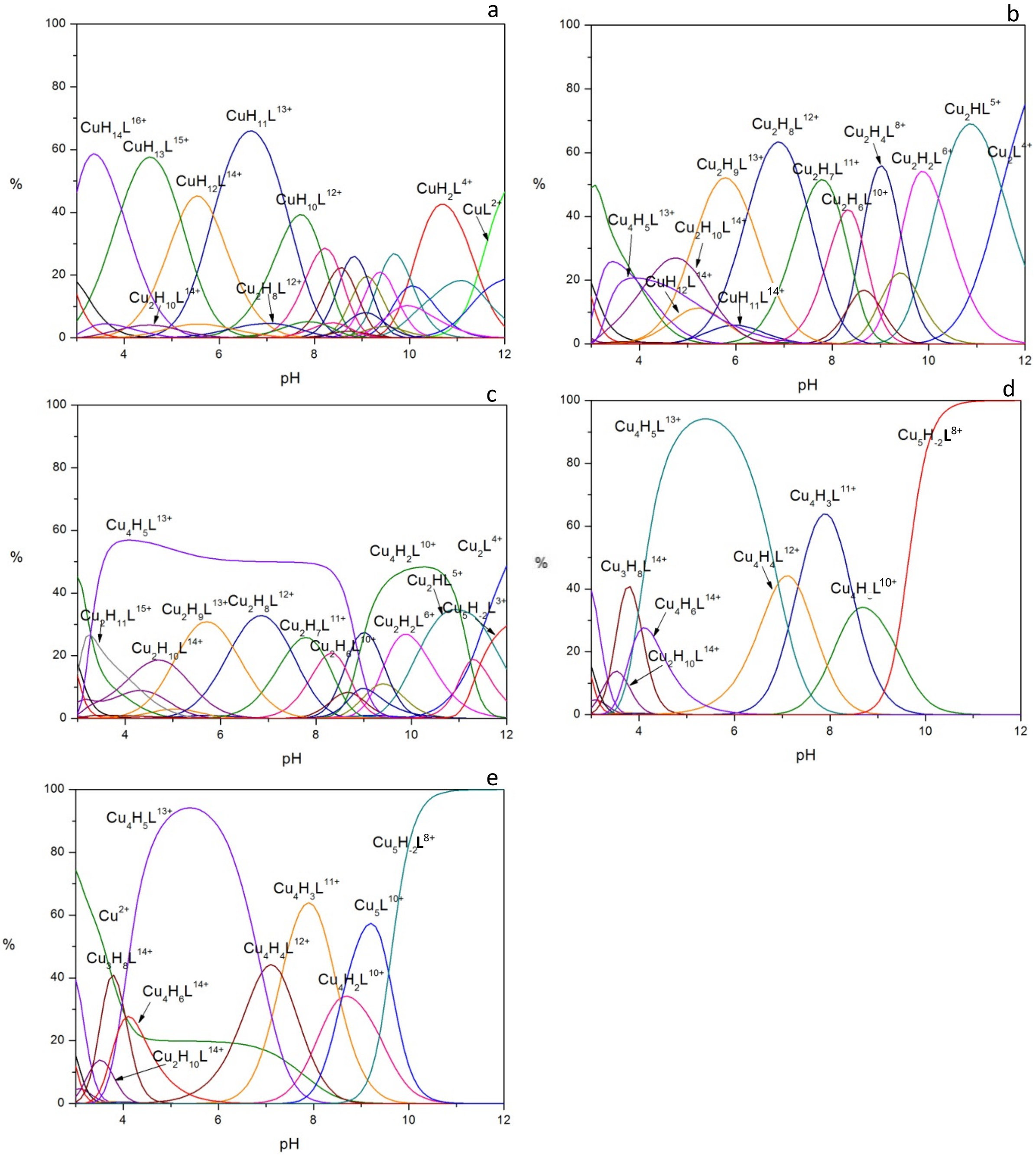

Figure S1. Distribution diagrams of the Cu^{2+} complexes of L2. [L2] = 1×10^{-3} M, [Cu^{2+}] = [L2] (a), [Cu^{2+}] = 2[L2] (b), [Cu^{2+}] = 3[L2] (c), [Cu^{2+}] = 4[L2] (d), [Cu^{2+}] = 5[L2] (e).

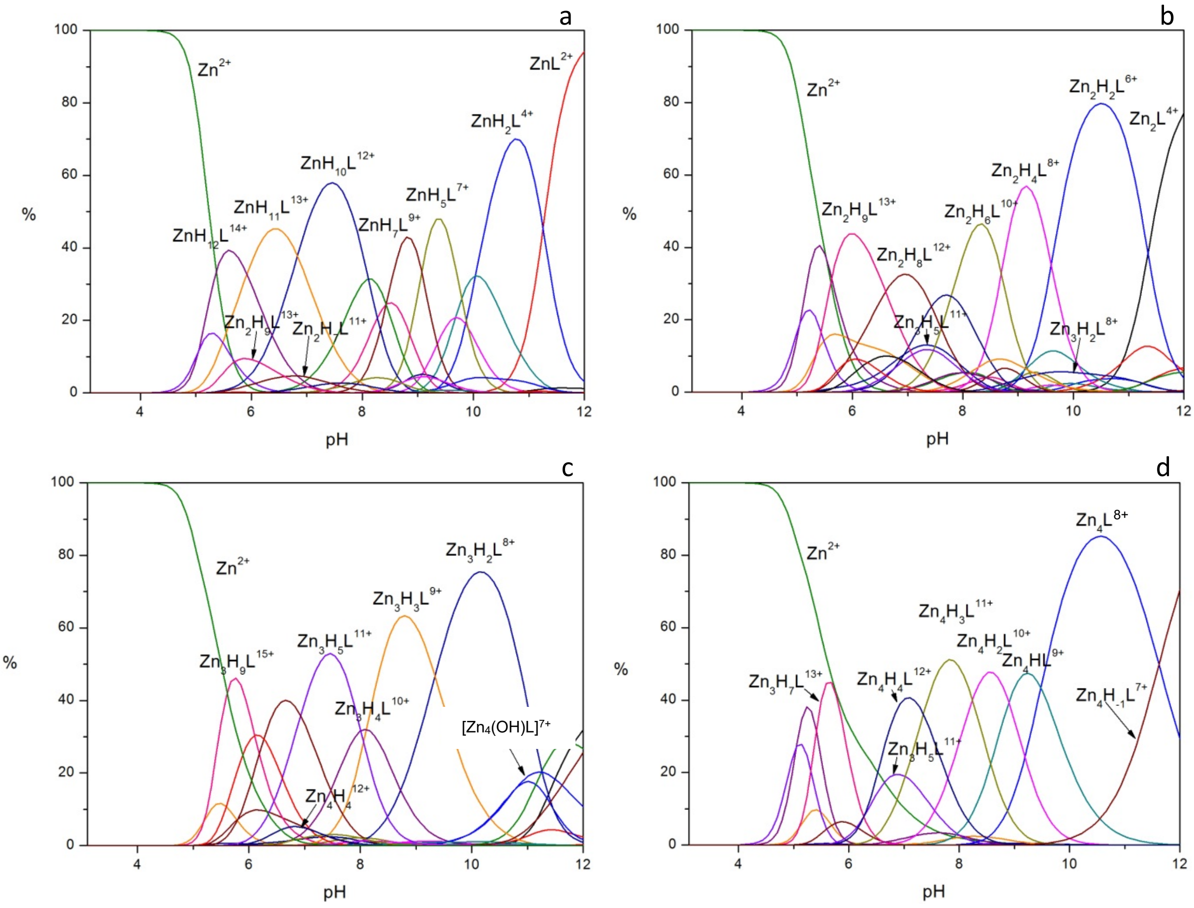

Figure S2. Distribution diagrams of the Zn^{2+} complexes of L2. [L2] = 1×10^{-3} M, [Zn^{2+}] = [L2] (a), [Zn^{2+}] = 2[L2] (b), [Zn^{2+}] = 3[L2] (c), [Zn^{2+}] = 4[L2] (d).

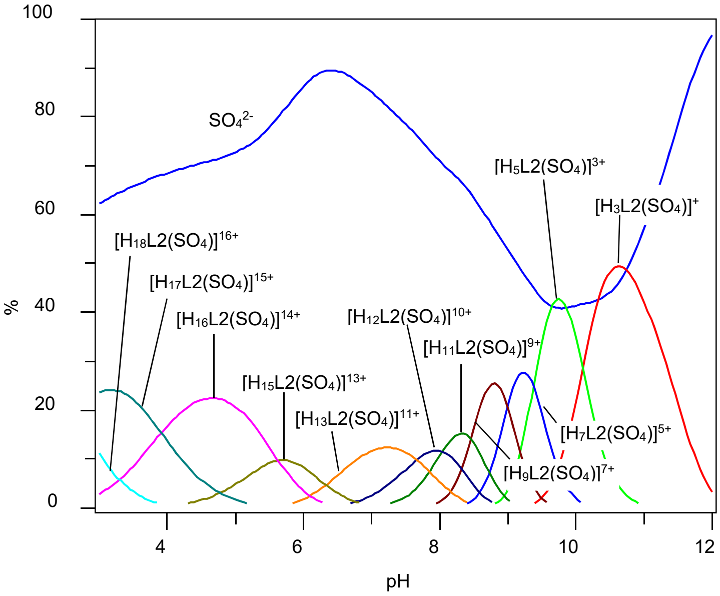

Figure S3. Distribution diagrams of the anion complexes formed by L2 with SO_4^{2-}. [L2] = [SO_4^{2-}] = 1×10^{-3} M.

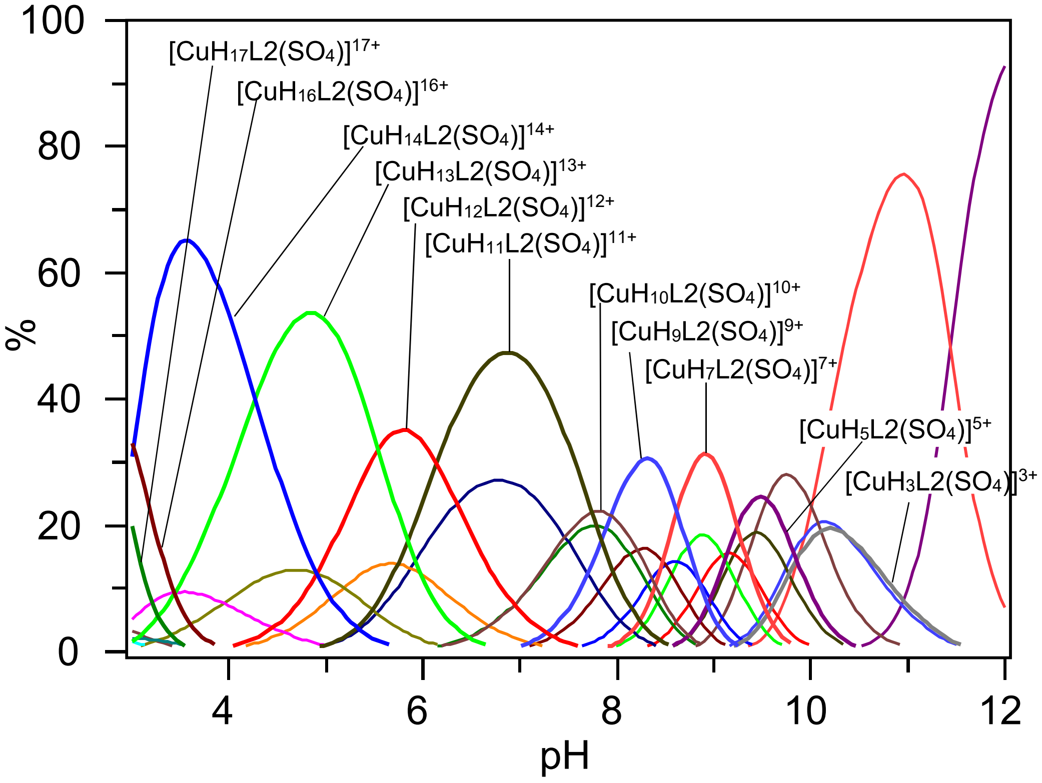

Figure S4. Distribution diagrams of the ion-pair complexes formed by L2 with Cu^{2+} and SO_4^{2-} complexes of L2. [L2] = [Cu^{2+}] = [SO_4^{2-}] = 1×10^{-3} M.
